# Percutaneous Endoscopic Lumbar Discectomy Assisted by O-Arm-Based Navigation Improves the Learning Curve

**DOI:** 10.1155/2019/6509409

**Published:** 2019-01-10

**Authors:** Shengxiang Ao, Junlong Wu, Yu Tang, Chao Zhang, Jie Li, Wenjie Zheng, Yue Zhou

**Affiliations:** Department of Orthopaedics, The Second Affiliated Xinqiao Hospital of Army Medical University, Chongqing 400037, China

## Abstract

**Objective:**

There is a steep learning curve with traditional percutaneous endoscopic lumbar discectomy (PELD). The aim of this study is to assess the safety and efficacy of PELD assisted by O-arm-based navigation for treating lumbar disc herniation (LDH).

**Methods:**

From September of 2017 to January of 2018, 118 patients with symptomatic LDH were enrolled in the prospective cohort study. The patients undergoing PELD with O-arm-based navigation technique were defined as group A (58 cases), and those undergoing traditional X-ray fluoroscopy method were defined as group B (60 cases). We recorded the operation time, cannula placement time, radiation exposure time, visual analog scale (VAS), Oswestry Disability Index (ODI), and Macnab criteria score of the 2 groups.

**Results:**

The average operation time (95.21 ± 19.05 mins) and the cannula placement time (36.38 ± 14.67 mins) in group A were significantly reduced compared with group B (operation time, 113.83 ± 22.01 mins, P<0.001; cannula placement time, 52.63 ± 17.94 mins, P<0.001). The learning curve of PELD in group A was steeper than that in group B and was lower in the relatively flat region of the end. There were significant differences of the clinical parameters at different time points (VAS of low back, P < 0.001; VAS of leg, P < 0.001; and ODI, P < 0.001). The VAS scores for low back pain and leg pain improved significantly in both groups after surgery and gradually improved as time went by. No serious complication was observed in any patients in either group.

**Conclusion:**

The study indicated that PELD assisted by O-arm navigation is safe, accurate, and efficient for the treatment of lumbar intervertebral disc herniation. It reshaped the learning curve of PELD, reduced the difficulty of surgery, and minimized radiation exposure to surgeons. This study was registered at Chinese Clinical Trail Registry (Registration Number: ChiCTR1800019586).

## 1. Introduction

Minimally invasive spine surgical techniques are constantly developing and progressively becoming common techniques for treating lumbar disk herniation (LDH) [1, 2]. Particularly, percutaneous endoscopic lumbar discectomy (PELD) could have less muscular injury and bleeding, less scar formation within the spinal canal, and shorter hospital day, compared with open discectomy [3, 4]. Therefore, PELD has been widely used in lumbar discectomy surgery.

Nevertheless, the traditional PELD technique has a steep learning curve [5], needing surgeons' tough training to overcome it. Design and intraoperative application on the proper trajectory of the puncture for foraminoplasty are highly experience- and technique-demanding. Some cases with high iliac crest or severe migration would magnify the difficulty of puncture, even for skilled surgeons [6, 7]. Reducing the operating difficulty, increasing the accuracy of puncture, and reducing the radiation exposure to both patients and medical staff are the common goals shared by every surgeon.

Along with the development of medicine and technology, navigation has been applied in spine surgery [8]. Under image guidance systems, surgeons can get a 3-dimensional (3D) anatomy structure of spine or the multiplanar imaging reconstruction, and surgical instruments can be tracked in real time for 3D space. Previous studies have described successful navigation-assisted surgery in the cervical vertebrae, which is a safe and effective option for cervical radiculopathy [9]. However, there were few studies published about O-arm-based navigation in PELD. The feasibility, security, and accuracy of navigation in lumbar are rarely reported.

In this study, we present a surgical technique of PELD assisted by an O-arm-based navigation system and explore the learning curve and clinical outcomes between navigation and non-navigation group in prospective consecutive case series of LDH.

## 2. Materials and Methods

### 2.1. Patients

From September of 2017 to January of 2018, 118 patients with symptomatic LDH received PELD by two surgeons were enrolled in the study. For reducing the experimental bias, the two junior surgeons were blinded to the study. They did not know the purpose and specifics of this study. Both of the surgeons had 4-year rich surgical experience in conventional open spinal surgery with the same medical background, and both of them could complete microendoscopic discectomy (MED) independently. Before conducting PELD on their own, they had been trained systematically for several weeks by the same senior surgeon, using the same method, including 3 PELD cases of hand-holding practical teaching.

The inclusion criteria were (1) age≥18 and ≤70 years; (2) typical clinical symptoms and signs of mono-radiculopathy LDH; (3) concordant imaging evidence of single LDH (limited to L3-4, L4-5, or L5-S1), such as computed tomography (CT) and magnetic resonance imaging (MRI); and (4) conservative therapy for at least 3 months before surgery. The exclusion criteria were (1) serious underlying disease or mental illnesses; (2) severe central stenosis, cauda equina syndrome, spinal instability, active infection, and serious calcified fragments; (3) previous lumbar treatment with spinal surgery, ozone intervention, or radiofrequency ablation; and (4) unwilling or unable to participate in treatment and complete follow-up.

The patients undergoing PELD with O-arm-based navigation technique were defined as group A (58 cases), and those undergoing traditional X-ray fluoroscopy method were defined as group B (60 cases). The mean follow-up period was 9 months and all patients completed at least 7 months of follow-up.

This prospective clinical contrast study was approved by the Medical Ethics Committee of the Second Affiliated Hospital of Army Medical University and it was registered at Chinese Clinical Trail Registry (Registration Number: ChiCTR1800019586). All patients had signed consent forms before the surgery.

### 2.2. Surgical Tools

The O-arm and computer-assisted navigation system (O-arm Surgical Imaging System and Stealth-Station; Medtronic, Inc., Minneapolis, Minnesota, USA), the spine transforaminal endoscope system (TESSYS instrument system; Joimax, Inc., Irvine, California, USA), the patented specially designed ZESSYS double-cannula instrument (Bosscom, Inc., Chongqing, China) for targeted foraminoplasty, and tip-flexible electrode bipolar radiofrequency system (Elliquence LLC, Baldwin, New York, USA) were used in PELD.

### 2.3. Surgical Technique

#### 2.3.1. O-Arm-Based Navigation (Group A) Surgical Procedure

**Figure 1:**
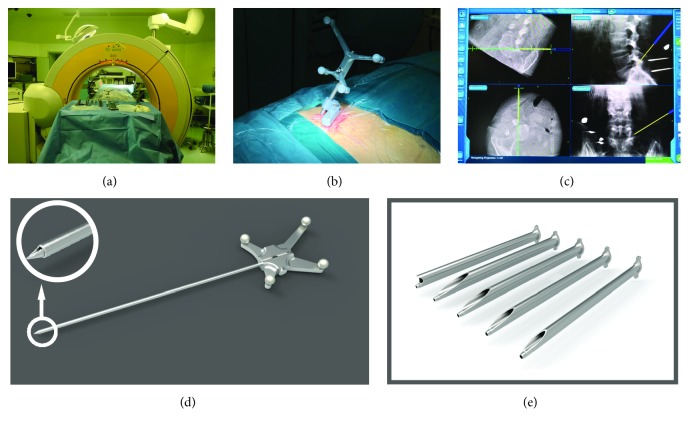
(a) Intraoperative 3D navigation system of O-arm. (b) The reference frame was fixed on the contralateral iliac crest. (c) With the aid of the real-time navigation, the entire puncture trajectory could be designed accurately and easily. (d) The trocar-like puncture probe for navigation. (e) In the dual-cannula (ZESSYS), the thinner cannula could contain a guide Kirschner wire for fixation and larger cannula for bony abrasion by bone drill/trephine.

The patient was placed prone on the radiolucent table. The O-arm Surgical Imaging System and Stealth-Station (Medtronic, Inc., Minneapolis, Minnesota, USA) were used for intraoperative stereotactic navigation (Figure 1(a)). After local anesthesia by 0.5% lidocaine, the reference frame was fixed on the contralateral iliac crest, using two Kirschner wires of 2.0mm diameter (Figure 1(b)). An intraoperative CT scan and 3D image were obtained by the O-arm with a medium dose (13s) of irradiation to reduce the radiation exposure to patient. The CT images data were rapidly transferred to the navigation system. Then the surgeon could get the multiplanar imaging reconstructions in both the axial and sagittal planes, traditional X-ray-like anteroposterior and lateral views, and even 3D image of the lumbar spine. And the surgeon could choose any images above, depending on his/her own habit. The final step of navigation preparation was the registration of surgical instruments, which could be tracked in real time. The entire procedure including reference frame fixation, scan, image transfer, and tools registration could take less than 10 minutes.

In navigation image, the tip of probe could be extended virtually along itself. With the aid of the sagittal reconstructions aimed at tip of superior articular process (SAP) and the axial views pointing over the anteriolateral margin of facet joint, the entire puncture trajectory targeted at tip of SAP could be designed accurately and proper skin entry point was selected easily (Figure 1(c)). Possible bony obstruct including high iliac crest and hypertrophic transverse process can be easily avoided in navigation views.

The entire surgery was performed under local anesthesia and optional narcotic sedation. A 0.7 cm incision was then made in the skin. A total amount of 15-30 mL of 0.5% lidocaine was infiltrated in the puncture trajectory through the trocar-like puncture probe (Figure 1(d)). With the help of navigation, when the probe was advanced docking on the lateral aspect of facet joint, it could easily slide into foramen along the anterior aspect of SAP. A 2 mm rod or Kirschner wire was introduced into foramen through the trocar probe and was slightly hammered to fix itself on the posterior aspect of the distal vertebra. After the sequential dilation, a patented double-cannula device named ZESSYS (Figure 1(e)) specially designed for navigation was then introduced to dock on the lateral aspect of facet joint.

The ZESSYS targeted foraminoplasty instrument offered variable options of facet-cutting amount and adjustable foraminoplasty site. Furthermore, the Kirschner wires in smaller tube provided a fixed pivot to avoid accidental instrument sliding during facet-cutting on facet joint. Under real-time navigation views guidance, the optimal foraminoplasty trajectory on SAP for intracanal exposure and neurological decompression based on different clinical needs could be easily designed and obtained. Depth of reamer/bone drill could also be easily monitored under navigation guidance to avoid possible intracanal neurological element injury.

After proper foraminoplasty, the working canal was then inserted through dilator. Further operation was performed under visual control of a 5.9 mm endoscope and continuous fluid flow with 0.9% saline solution. Discectomy and nerve root decompression were performed as routine PELD procedure [10].

#### 2.3.2. Traditional X-Ray Fluoroscopy (Group B) Surgical Procedure

This procedure was performed as routine PELD with the standard TESSYS technique [10].

### 2.4. Clinical Assessment

Basic patient information, such as gender, age, body mass index (BMI), follow-up time, and LDH location, was recorded. We also recorded operation time (for the navigation group, it included the time of the O-arm surgical imaging setup procedure), intraoperative cannula placement time, and radiation exposure time. The intraoperative cannula placement time was defined as the duration between the first puncture and the final placement of working cannula (for the navigation group, it was calculated from the first puncture of the reference frame fixation). The radiation exposure time of Group B was obtained from the G-arm at the end of each procedure. We measured Macnab score (excellent, good, fair, poor) at 7 months' follow-up, which was used to assess patients' satisfaction and functional outcomes, in addition to preoperative and postoperative visual analogue scale (VAS) (0-10) of leg and low back pain and Oswestry Disability Index (ODI) at the time points of 1 day, 3 months, and 7 months after surgery and subsequently if required.

### 2.5. Statistical Analysis

Statistical software SPSS18.0 (SPSS, Inc., Chicago, IL, USA) was used to conduct the statistical analysis. The differences of the clinical outcomes (VAS and ODI) between the 2 groups and the changes over time in each group were identified via repeated-measures analysis of variance generally. LSD test was used to confirm further the changes at different time points in the same group, and Student's t-test was used to confirm further the differences between the 2 groups at the same time points. The difference of measurement data that were demonstrated as the mean ± standard deviation (SD), such as age, BMI, operation time, and cannula placement time, was assessed by Student's t-test. Ranked data such as Macnab criteria were detected by Mann-Whitney U test. Calculator information, such as gender and level ratio, was analysed by Chi-square test. Statistical significance was set at a P value of <0.05.

The learning curve was fitted with 11 different curve estimation regression models (linear, logarithmic, inverse, quadratic, cubic, power, compound, S-curve, logistic, growth, and exponential) by SPSS 18.0, where “y” is the operative time and “x” is the chronological operation case number. The regression model of learning curve was finally set depending on the highest R value among the 11 related plots and being consistent with the actual situation.

## 3. Results

### 3.1. Patient Demographic Characteristics

One hundred and eighteen patients (group A, 58 patients; group B, 60 patients) who underwent PELD between September of 2017 and January of 2018 were consecutively enrolled in this study. No significant differences (P > 0.05) were detected in the preoperative demographics between group A and group B (Table 1).

### 3.2. Clinical Outcomes

As shown in Table 2, the average operation time (95.21 ± 19.05 minutes) and the cannula placement time (36.38 ± 14.67 minutes) in group A were significantly shorter compared with group B (operation time 113.83 ± 22.01 minutes, P < 0.001; cannula placement time, 52.63 ± 17.94 minutes, P < 0.001). The radiation exposure time of group A was 13 seconds as the O-arm's setting, and in group B it was 53.47 ± 9.42 seconds.

Depending on the results of repeated-measures analysis of variance, there were significant differences of the clinical parameters at different time points (VAS of low back, P < 0.001; VAS of leg, P < 0.001; and ODI, P < 0.001). The VAS scores for low back pain and leg pain and ODI scors improved significantly in both groups after surgery and gradually improved as time went by (Table 3). However, there were no significant differences between the 2 groups (VAS of low back, P = 0.469; VAS of leg, P = 0.706; and ODI, P = 0.354). The excellent and good rates of Macnab criteria were 87.93% in group A and 83.33% in group B. We found no significant difference in Macnab criteria between group A and group B (P = 0.249).

### 3.3. Learning Curve

**Figure 2:**
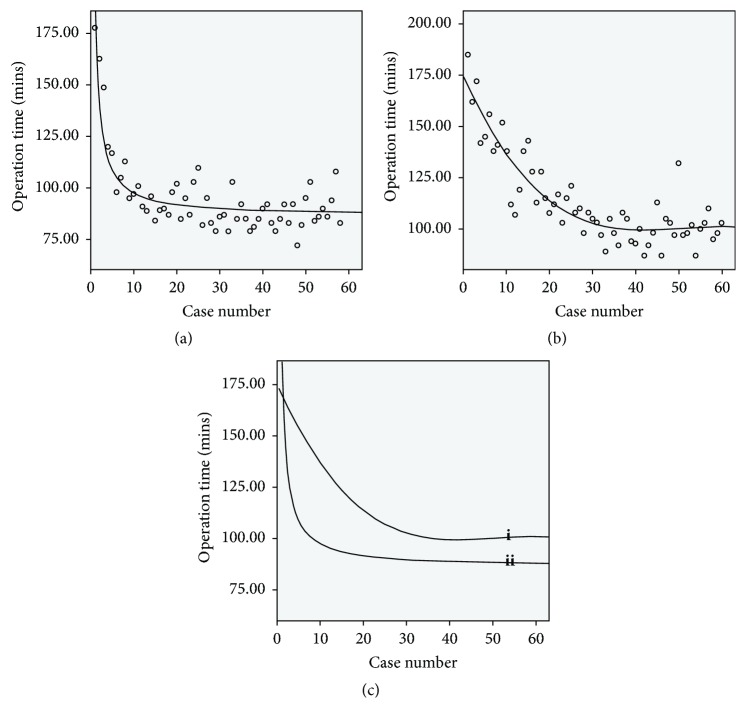
The learning curve of PELD. (a) Group A: y = 86.21+112.26/x. (b) Group B: y = 174.483-4.712x+0.0966x^2^-0.000641x^3^. (c) Draw Group A curve (ii) and Group B curve (i) in the same coordinate.

In group A, the learning curve was characterized using an inverse regression analysis (y = 86.21+112.26/x, R^2^ = 0.765, P < 0.001). As demonstrated in Figure 2(a), increasing case number was associated with fast decreasing operative time, and the curve tended to be stable in the end, where y = 95. Depending on the equation, y = 86.21+112.26/x, we deduced that from case 13 (x = 12.77, where y = 95) the doctor gradually reached a proficient phase.

In group B, the learning curve was characterized using a cubic regression analysis (y = 174.483-4.712x+0.0966x^2^-0.000641x^3^, R^2^ = 0.804, P < 0.001). As demonstrated in Figure 2(b), increasing case number was associated with slowly decreasing operative time, and the curve tended to be stable in the end with a relatively flat region, where y = 102. Depending on the equation, y = 174.483-4.712x+0.0966x^2^-0.000641x^3^, we deduced that from case 32 (x = 31.26, where y = 102) the doctor gradually reached a proficient phase.

As demonstrated in Figure 2(c), the learning curve of PELD in group A was steeper than that in group B and was lower in the relatively flat region of the end.

### 3.4. Operation Complications

There were one case of recurrence and five cases of pain symptom remnants in group A, whereas there were two cases of recurrence and seven cases of pain symptom remnants in group B. No major complications including dura tear, spinal instability, vascular injury, surgical infection, or serious nerve injury were observed. Only one patient in group B suffered a slight nerve injury after the surgery at the L5-S1 level. He received conservative treatments such as neurotrophic drug and medium frequency pulse electrotherapy and recovered completely during the follow-up period. All included patients are still in long-term follow-up, without lost follow-up case.

## 4. Discussion

The traditional PELD poses great challenges to surgeons because the percutaneous transforaminal approach requires a proper point of entry and accurate puncture trajectory [10]. With conventional 2D fluoroscopy-guided discectomy, surgeons need their rich experience to complete an accurate puncture, which is also a challenge of anatomy and spatial imagination ability. It leads to a steep learning curve of PELD.

Reducing the operating difficulty and the risk of damage to the nerve root and vital tissue in puncture, simplifying the operating, and reducing the radiation exposure to both patients and medical staff, all the above are surgeons' constant goal. Lee et al. [11] found that proper pre-PELD training and patient selection may make the learning curve more acceptable. Chaichankul et al. [12] found that, because of the difficulty of PELD, the amount of surgical volume has an influence in the improvement of the effect of discectomy. Fan et al. [13] designed a mechanical navigation tool to reshape the learning curve of PELD.

**Figure 3:**
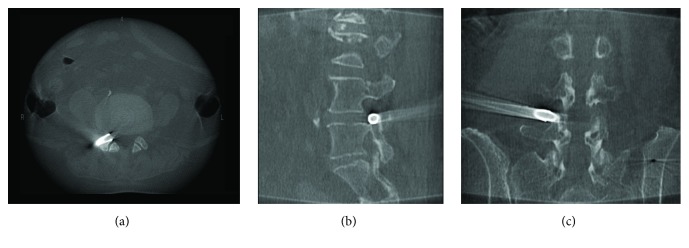
The axial (a), sagittal (b), and coronal (c) images of intraoperative CT re-scan during foraminoplasty.

**Figure 4:**
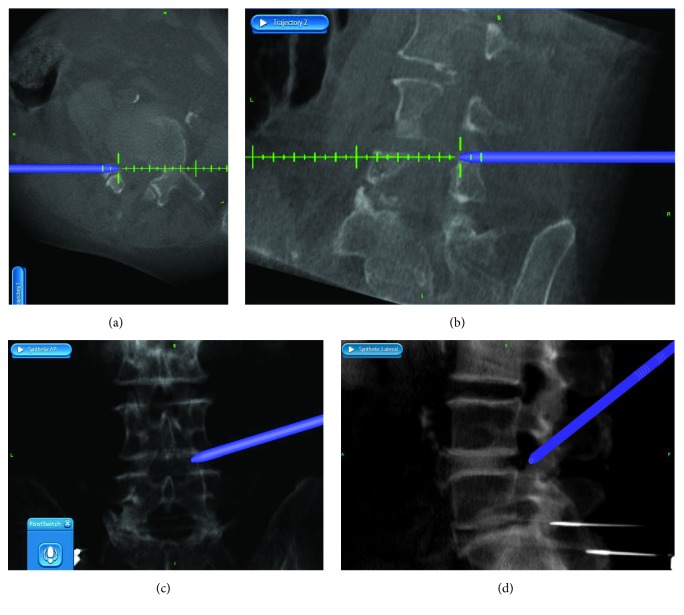
The axial (a) and sagittal (b) reconstructed images and anteroposterior (c) and lateral (d) composite images of intraoperative navigation during foraminoplasty.

**Figure 5:**
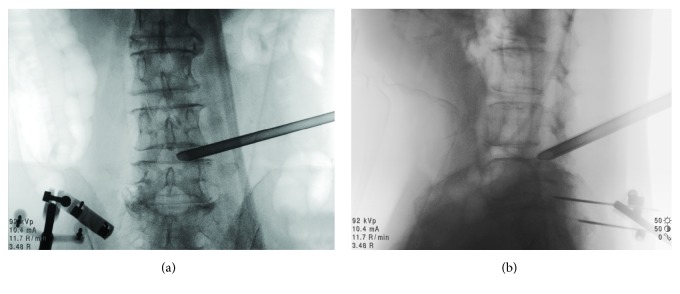
The anteroposterior (a) and lateral (b) fluoroscopy views of intraoperative radiograph after the working cannel placed.

Along with the development of medicine and technology, computer-assisted 3D navigation has begun to be used in spine surgery [8, 14]. 3D navigation enables the surgeon to visualize the correct puncture trajectory at all times. Previous study reported that the reference frame was fixed on a spinous process, following the manufacturer's recommendations [15]. However, it led to unnecessary injury to spine. In our cases, the reference frame was fixed on the contralateral iliac crest. In order to verify the accuracy of navigation and keep safe when the reference frame was not attached on spine, during intraoperative foraminoplasty, we had re-scanned the surgical site in the preliminary clinical application of 10 cases before this prospective cohort study. The intraoperative CT re-scan by O-arm showed trephine cut off the anterior aspect of SAP accurately and it was safe to conduct foraminoplasty (Figure 3). At the same time, the intraoperative navigation images (Figure 4) were perfectly matched with intraoperative CT re-scan (Figure 3). After the working cannel was inserted, we re-scanned again by intraoperative radiograph of O-arm, to make sure of the ideal placement of working cannel (Figure 5). The re-scan results showed the accuracy loss is acceptable for this technique. In our study, PELD assisted by O-arm-based navigation is completely feasible in L3-4, L4-5, and L5-S1. The preoperative pain and functional scores were significantly improved at all time points after the PELD with navigation. There are no major complications observed. The excellent and good rates of Macnab criteria were 87.93% in navigation group.

The procedure from the initial punctures to the final placement of working cannel is the most difficult and critical part of the surgery [16]. Even the initial selection of entry point is also a huge challenge, because the surgeon needs to individually choose the correct distance from the midline of the spinous process based on the specific conditions of patients, such as height, weight, and anatomic feature. Choi et al. [17] reported a single-center experience of 10,228 cases, which showed that nonideal puncture and working cannel position were important factors leading to unsuccessful PELD. Nevertheless, O-arm-based navigation technique resolves the critical problem easily and simplifies the method of puncture. In this study, there was a significant reduction in the operation time and cannula placement time of navigation group. The learning curve of PELD in group A was steeper than that in group B, and the whole curve of navigation group was under that of the conventional group. With navigation technique, it took about 13 cases to arrive at a relatively stable proficiency condition, whereas it took about 32 cases with conventional technique. It should be noted that the steeper learning curve might not be a bad thing because beginners could master PELD technique faster with standard exercise on fewer patients [18, 19]. These positive results showed that the O-arm-based navigation system could help surgeons break the technique barriers brought by puncture and foraminoplasty portion and then reduce the technique difficulties of PELD.

In the first few cases of the navigation group, the operation time went far beyond the average time (95 minutes). It is mainly due to the nonproficiency of computer operation and poor cooperation with the technician. After a transient adaptation, the whole procedure including reference frame fixation, scan, image transfer, and registration can take less than 10 minutes. The average operation time of navigation group was about 95 minutes, which is consistent with or less than other previous studies [11, 12, 19, 20].

In conventional procedure, puncture, foraminoplasty and placement of the cannula were performed by trial-and-error manner, which is challenging for even skilled spine surgeons [10]. Also, it is the most likely part to injure nerve. The ZESSYS foraminoplasty instrument is inserted via a Kirschner wire in the long thinner cannula of double-cannula, which plays the role of isolation and protection. Precise foraminoplasty under 3D Navigation guidance combined with double-cannula technique protection excludes the exiting nerve root from the working zone of the trephine providing definite neurological safety.

ZESSYS, the targeted foraminoplasty instrument specially designed for navigation procedure, meanwhile offered variable options of facet-cutting amount and adjustable foraminoplasty site. During the procedure of foraminoplasty, trephine was used to move anterior aspect bone of SAP. If driven by hand without any fixation, the trephine was easy to drift. However, in the dual-cannula, the thinner cannula could contain a guide Kirschner wire for fixation and larger cannula for bony abrasion by bone drill/trephine. The Kirschner wires in the smaller tube would be fixed at the posterior aspect of the distal vertebra providing a fixed pivot to avoid accidental instrument sliding during facet-cutting on irregular lateral shape of facet joint. Furthermore, the double-cannula could be rotated by the center of fixed Kirschner wire, and then the larger cannula could be easily docked on the SAP for target foraminoplasty.

The radiation exposure to both patients and medical staff is a great concern in spine surgery [21, 22]. Navigation-assisted fluoroscopy will not prevent exposure to the patient since they must remain in the radiation field during image acquisition. Fortunately, radiation exposure to patients is limited to the procedure itself. Unless they are undergoing multiple procedures involving fluoroscopy, their risk has been negligible. In a recent experimental study of radiation exposure to the fetus, it was estimated that at least 35 minutes of fluoroscopy would be needed for the induction of radiation related effects [23]. However, the medical staff suffered cumulative radiation exposure during every surgery, especially for the spine surgeons. When comparing radiation exposure experienced by a spine surgeon to other orthopedic subspecialties, a spine surgeon sees 50 times the lifetime radiation dose compared to that of a hip surgeon [24]. The authors in [25] have demonstrated that, in the case of the O-arm system, there exists little to no scatter at distances beyond approximately 4 m. Therefore, technically, there is minimal to no radiation exposure to the surgeons, which reduces the harm to medical staff.

## 5. Conclusions

The study indicated that PELD assisted by O-arm navigation is safe, accurate, and efficient for the treatment of lumbar intervertebral disc herniation. It reshaped the learning curve of PELD, reduced the difficulty of surgery, and minimized radiation exposure to surgeons.

## Figures and Tables

**Table 1:** Patient demographics of group A and group B.

	**Group A**	**Group B**	**P Value**
**Gender (male: female)**	39:19	37:23	0.527
**Age (years)**	45.19 ± 13.63	42.43 ± 12.36	0.252
**BMI (kg/m** ^**2**^ **)**	24.42 ± 4.14	23.76 ± 3.80	0.372
**Follow-up Time (months)**	8.90 ± 1.46	9.02 ± 1.48	0.658
**Levels**			0.655
L3-4	2	2	
L4-5	26	22	
L5-S1	30	36	

BMI: body mass index.

**Table 2:** Comparison of clinical outcomes in group A and group B.

	**Group A**	**Group B**	**P Value**
**Operation time (mins)**	95.21 ± 19.05	113.83 ± 22.01	<0.001
**Cannula placement time (mins)**	36.38 ± 14.67	52.63 ± 17.94	<0.001

**Table 3:** Comparison of follow-up outcomes in group A and group B.

	**Group A**	**Group B**	**P Value**
**VAS of low back**			
Preoperative	5.41 ± 2.24	4.98 ± 2.02	0.275
1 day	2.38 ± 1.40*∗*	2.50 ± 1.36*∗*	0.636
3 months	2.00 ± 1.08 *∗*	1.85 ± 1.15*∗*	0.466
7 months	1.53 ± 0.96*∗*	1.38 ± 0.96*∗*	0.394
**VAS of leg**			
Preoperative	6.14 ± 1.86	5.67 ± 1.50	0.132
1 day	2.07 ± 1.21*∗*	2.30 ± 1.25*∗*	0.311
3 months	1.83 ± 1.11*∗*	1.93 ± 1.06*∗*	0.597
7 months	1.33 ± 1.11*∗*	1.22 ± 1.14*∗*	0.594
**ODI**			
Preoperative	58.97 ± 17.79	55.80 ± 14.66	0.293
1 day	19.83 ± 6.59*∗*	18.87 ± 5.54*∗*	0.392
3 months	16.86 ± 4.76*∗*	16.30 ± 4.97*∗*	0.531
7 months	12.83 ± 5.83*∗*	13.37 ± 6.88*∗*	0.648
MacNab criteria†			
7 months	35:16:6:1	30:20:8:2	0.249

*∗*Compared with preoperative, P<0.05.

†Excellent: good: fair: poor.

VAS: visual analog scale, ODI: Oswestry Disability Index.

## Data Availability

The data used to support the findings of this study are available from the corresponding author upon request.
